# The current status, relevance and impact of the sense of gain and social participation among Chinese youth: A study based on the CSS2021 database

**DOI:** 10.1371/journal.pone.0313950

**Published:** 2024-12-20

**Authors:** He Yang, Changan Li, Liujun Ye

**Affiliations:** School of Government, University of International Business and Economics, Beijing, China; Zhejiang Gongshang University, CHINA

## Abstract

Youth symbolize the future and promise of the country. The contributions of youth are integral to the comprehensive development, the social participation of youth is crucial for their holistic development and the betterment of society. As the people’s sense of gain has evolved into a fundamental aspect of high-quality development in the new era of China, investigating the influence mechanism of the youth’s sense of gain on social participation holds significant historical and practical relevance. This study utilizes the CSS2021 database to conduct empirical analysis, centered on three pivotal dimensions, the youth’s sense of political gain, economic gain, and people’s livelihood gain, this study explores their influence on social participation across affairs, rights protection, and people’s livelihood, and the study further delves into the specific impact mechanisms through which the youth’s sense of gain influences social participation. The results distinctly demonstrate that the sense of gain within the youth group significantly influences the enthusiasm for social participation. The study reveals specific associations: the sense of economic gain negatively correlates with social participation in affairs and rights protection, while the sense of people’s livelihood gain demonstrates a positive correlation with rights protection. Additionally, a noteworthy positive correlation exists between political gain and social participation. Consequently, there is a suggestion that the government could better understand the core demands of young individuals, thereby fostering increased enthusiasm for their social participation. Leveraging the active engagement of young people could significantly contribute to enhancing the government’s governance effectiveness.

## Introduction

The youth are not just a symbol of hope for the future but also the dynamic force propelling the nation’s progress forward. Their engagement in societal and economic activities, coupled with their creative and innovative spirit, has historically played a critical role in advancing civilization. In the contemporary era, the contributions of young people are more crucial than ever. They are at the helm of driving productivity improvements, particularly by pioneering technological innovations and leading the charge in the digital revolution. Their expertise in high-tech fields and their ability to navigate and shape the rapidly evolving landscape of the internet underscore their role as key players in modern society. Moreover, today’s youth are instrumental in aligning the nation with global standards. They are open to new ideas, readily embracing diverse perspectives and integrating these into the local context, thereby fostering societal progress. This generation’s willingness to explore and implement new concepts positions them as the vanguards of change, bridging the gap between tradition and modernity, and ensuring that the country remains competitive on the global stage. However, despite the remarkable economic growth and the substantial improvements in living standards that China has experienced, the nation continues to face significant challenges. The rapid development has brought to light several pressing issues, such as the widening income gap, the growing divide between urban and rural areas, and the uneven pace of development across different regions. These disparities have led to imbalances that threaten social harmony and the overall cohesion of society. Addressing these challenges is critical to ensuring that the benefits of growth are shared more equitably across all segments of the population. It is essential to implement strategies that not only promote balanced regional development but also narrow the income gap and bridge the urban-rural divide. These efforts are vital for mitigating social tensions and ensuring long-term, sustainable progress. Finding effective solutions to these complex issues will be imperative for sustaining the nation’s momentum and ensuring a prosperous and harmonious future for all [1].

At this stage in China’s development, the people’s sense of gain has become a cornerstone of high-quality development in the new era. It reflects the tangible and intangible benefits that individuals perceive as a result of economic policies, social programs, and reforms, encompassing not only economic prosperity but also broader social well-being, including security, fairness, and satisfaction with public services. This sense of gain has become a crucial benchmark for assessing government effectiveness. It serves as a direct indicator of how well the government meets the needs and aspirations of its people, providing a more people-centered perspective on the outcomes of societal reforms. The ability to enhance the people’s sense of gain is increasingly seen as a testament to the government’s governance capabilities and its commitment to building a more equitable and prosperous society [2, 3]. The level of citizens’ social participation is a crucial indicator of healthy social development. When individuals are actively engaged in their communities, it reflects a strong, dynamic society. This willingness to participate is closely tied to their sense of gain—the perception that they benefit from and contribute positively to society. When people feel that their lives are improving and that they have a stake in their community, they are more likely to participate in social, economic, and political activities. Thus, fostering a sense of gain among citizens is essential for encouraging active participation, which is key to sustaining social progress [4, 5].

The enhancement of youth social participation has emerged as a key priority for governments, recognizing the vital role that young people play in shaping the future of society. However, there is a noticeable lack of academic research exploring the connection between the youth’s sense of gain and their active involvement in social participation. This gap in the literature highlights the need for a deeper understanding of how young people’s perceptions of their well-being—encompassing political, economic, and livelihood aspects—influence their willingness to engage in various social activities and civic duties. This relationship is crucial for developing effective policies that encourage greater youth participation across different societal domains, such as community service, political engagement, and social advocacy. A comprehensive examination of how the youth’s sense of gain impacts their social participation can provide valuable insights for policymakers. By understanding these dynamics, policymakers can design targeted strategies that resonate with the needs and aspirations of young people, ultimately fostering a more vibrant and inclusive social fabric. Strategic interventions that enhance the youth’s sense of gain could serve as catalysts for increased social participation, leading to a more active and engaged youth population. This, in turn, would contribute to the development of a more cohesive and dynamic society, where young people feel empowered to take on leadership roles and drive positive change. As such, addressing the research gap in this area is not only important for academic inquiry but also for the practical goal of creating a more participatory and inclusive society.

## Literature review and research hypothesis

This section provides a comprehensive overview of the theoretical foundations relevant to the study, along with a review of existing literature on the sense of gain among youth and their social participation. The theoretical framework incorporates concepts from social justice theory, participatory governance theory, social cohesion theory, social exchange theory, social capital theory, and theories of civic engagement. These theories collectively illuminate the dynamics of youth engagement, emphasizing the importance of fairness, inclusion, and social connections in fostering a robust sense of gain. The literature review on the sense of gain among youth explores various dimensions, including how access to resources, opportunities, and equitable treatment influences young people’s perceptions of well-being and fulfillment. Previous studies highlight the critical role of social structures and institutional support in shaping these perceptions, particularly within the context of rapid social change in China. In parallel, the review of literature on youth social participation examines the factors that motivate young individuals to engage in civic activities. Research indicates that social participation not only enhances individual development but also contributes to community resilience and social cohesion. The interplay between social capital and youth engagement is particularly noteworthy, as it suggests that active involvement in social networks can lead to greater opportunities and collective benefits. Based on the theoretical underpinnings and existing literature, the research hypotheses are formulated. It is posited that a strong sense of gain among Chinese youth is positively associated with their level of social participation. Additionally, it is hypothesized that factors such as perceived fairness and inclusivity in societal structures significantly enhance their motivation to engage in civic activities. These hypotheses aim to bridge the gap between theoretical insights and empirical evidence, providing a foundation for further exploration of the interconnectedness between youth well-being and social participation.

### Theoretical foundations

The theoretical foundation of this study draws on several key theories to explain the dynamics between the sense of gain and social participation among Chinese youth. These theories offer insights into how individuals engage with society, how fairness and justice shape their experiences, and how social ties and collective action foster societal well-being.

Social justice theory provides a crucial lens through which to examine the sense of gain among Chinese youth. It emphasizes the importance of fairness, equity, and equal access to resources and opportunities [6]. In this context, "sense of gain" refers not just to material wealth but to a broader perception of well-being and equity in life opportunities. Chinese youth, as a demographic group, are particularly sensitive to issues of fairness, given the fast-paced changes in society and growing disparities in wealth and access to education, employment, and other resources. Social justice theory suggests that a perceived lack of fairness can lead to disengagement and dissatisfaction, while an equitable distribution of resources fosters a stronger sense of belonging and fulfillment. Participatory governance theory is essential in understanding the role of social participation. This theory argues that all societal groups, including youth, should be involved in decision-making processes to ensure that diverse perspectives are included and that outcomes are equitable [7]. For Chinese youth, participation in governance and civic activities, such as volunteerism or public consultations, reflects not only an engagement with public affairs but also a pathway to assert their needs and aspirations in a rapidly transforming society. The involvement of youth in such processes strengthens democracy and helps address social inequalities, as their voices become integral in shaping policies that directly affect their future. The absence of such participation, on the other hand, can result in a sense of marginalization. Social cohesion theory highlights the importance of inclusive practices in building strong, stable communities. It argues that when individuals feel they are part of a cohesive group, their sense of belonging and attachment to society grows, which in turn promotes stability and trust [8]. For Chinese youth, social cohesion is particularly important as they navigate the pressures of economic development, social change, and cultural expectations. Their engagement in social and communal activities—whether through digital platforms or local organizations—contributes to the bonding and bridging of social capital, thereby fostering a stronger sense of unity and shared purpose. Addressing inequalities and providing inclusive opportunities for youth engagement are therefore crucial to maintaining social stability.

Social exchange theory provides a behavioral understanding of why individuals engage in social participation. According to this theory, people are motivated by the potential for personal and social rewards [9]. In the context of Chinese youth, social participation—whether through volunteering, activism, or community events—is often seen as a means to gain social recognition, build networks, and accumulate valuable social and economic resources. This exchange of effort for perceived benefits, such as career opportunities or enhanced social standing, reinforces their commitment to social activities. Youth participation, in this light, is not merely altruistic but a calculated balance of benefits versus costs, where engagement is driven by a desire to improve both individual and collective outcomes. Social capital theory further elaborates on how social participation enhances both individual and collective well-being by fostering networks of relationships. For Chinese youth, these networks are critical in navigating a competitive environment where personal connections can be instrumental in gaining access to opportunities [10]. By actively participating in social groups, community projects, or online platforms, youth accumulate social capital, which can translate into practical benefits such as job prospects, mentorship, or social mobility. Moreover, social capital strengthens community ties, contributing to a more resilient social fabric, which benefits both individuals and society at large. Theories of civic engagement emphasize the significance of active involvement in public life as a means of fostering a sense of belonging and efficacy [11]. For Chinese youth, civic engagement offers a platform to express their views, challenge societal norms, and shape the future of their communities. Whether through political participation, volunteering, or advocacy, their active role in civic life reinforces their connection to society and provides a sense of agency. This sense of empowerment is particularly relevant for youth, as it contributes to their overall sense of gain, enhancing their mental and emotional well-being. Theories of civic engagement thus provide a comprehensive understanding of how youth participation contributes to both personal development and the broader social order.

The interplay of these theoretical frameworks—social justice, participatory governance, social cohesion, social exchange, social capital, and civic engagement—offers a nuanced understanding of the challenges and opportunities faced by Chinese youth. In a rapidly evolving society, Chinese youth are navigating the pressures of economic growth, social mobility, and cultural transformation, all of which affect their sense of gain and social participation. These theoretical insights reveal that youth engagement is not only vital for individual fulfillment but also for maintaining social stability and promoting long-term development. Addressing their needs for fairness, inclusion, and empowerment is therefore crucial in fostering a dynamic and responsive society.

### Research on youth’s sense of gain

The concept of the "sense of gain" is uniquely Chinese and remains underexplored in global studies. However, a similar idea, "social inclusiveness," is crucial for social development. Governments must prioritize all societal groups, especially vulnerable and marginalized ones. Inclusiveness and accountability are key to effective governance, ensuring fair representation and opportunities for all, which fosters social cohesion and equitable progress. Theoretical mechanisms supporting this include "social justice theory", which emphasizes the need for fairness and equal access to resources and opportunities. "Participatory governance theory" underscores the importance of involving all societal groups in decision-making processes to ensure diverse perspectives and equitable outcomes. "Social cohesion theory" highlights that inclusive practices strengthen community bonds and promote stability by addressing inequalities and fostering a sense of belonging. While the "sense of gain" is distinct to China, the global focus on "social inclusiveness" reflects these principles and underscores the importance of inclusive governance for societal advancement [2]. Especially in developing countries, governments play a crucial role in fostering inclusive development by addressing poverty, providing essential public services, ensuring social protection, and promoting employment [3]. The concept of the "sense of gain" encompasses both objective and subjective dimensions. Objectively, it pertains to the tangible or actual benefits perceived by individuals, comprising material and economic gains, access to rights, and sharing in the fruits of development. Subjectively, it extends to personal aspects like citizen self-realization and a sense of belonging within society [12]. The concept of the "sense of gain" operates on two levels: synchronic and diachronic. Synchronic refers to comparing current gains with anticipated future gains, emphasizing continuous improvement. The "sense of gain" also has absolute and relative dimensions. "Absolute gain" involves direct perceptions of material, economic benefits, and political rights, while "relative gain" arises from comparing one’s status or benefits with others or societal averages. This comparison helps individuals assess their sense of gain in relation to others in society [13]. The "sense of gain" differs from happiness and satisfaction by focusing on tangible, measurable benefits rather than subjective experiences. While happiness and satisfaction are personal and often lack objective measures, the "sense of gain" emphasizes quantifiable outcomes, giving it a more practical significance rooted in actual advantages [14].

The "sense of gain" is vital for driving reforms and building government trust. It enriches the evaluation of ideological reform by moving beyond GDP as the sole measure of development and ensuring a focus on qualitative aspects, not just input-output metrics [15]. The introduction of the "sense of gain" as a guiding principle in social reform serves to crystallize the ultimate goal of development endeavors. It acts as both a breakthrough and a foundational foothold in navigating and steering social reform initiatives [16]. Improving people’s sense of gain stands as a crucial priority to enhance government trust, given its profound impact on public perceptions [17]. To assess and enhance the sense of gain, measurement approaches include theoretical, real-world, and anticipatory dimensions. Evaluations consider income, equity, and sustainability both horizontally and vertically. Improving the sense of gain involves focusing on community governance, refining public services, strengthening legal frameworks, and incorporating it into government performance evaluations [18]. The academic community studies the sense of gain across various groups, including youth, teachers, farmers, and public service workers. Research focuses on enhancing the youth’s sense of gain, which affects social security, inclusion, empowerment, and freedom, as well as social cohesion. Key influencing factors include personal income, social equity, political engagement, values, satisfaction with social life, and societal trust. These elements collectively shape the sense of gain among youth [19, 20]. To enhance the sense of gain among youth, a dual approach is needed. The government should prioritize youth needs, understanding their evolving characteristics and focusing on key areas to boost their sense of gain. Additionally, improving social equity and justice is essential for increasing social inclusion and support for young people [21].

### Research on youth social participation

The sense of social participation reflects a societal consciousness and influences engagement choices and outcomes. It serves as a key indicator of a society’s vitality and development, originating from individuals’ social needs and their voluntary involvement in public affairs. This engagement demonstrates the dynamism and responsiveness of citizens and their contribution to the broader social fabric. Theoretical mechanisms underpinning this sense include "social exchange theory", which posits that individuals participate in social activities to gain personal and social rewards, balancing perceived benefits against costs. "Social capital theory" also plays a role, suggesting that social participation builds networks of relationships that enhance individual and collective well-being. "Theories of civic engagement" further highlight that active participation fosters a sense of belonging and efficacy, reinforcing societal involvement and contributing to social cohesion [4]. From the perspectives of social members’ roles, citizen needs, and state interests, social participation can be defined as the engagement in activities by citizens. These activities aim to address personal needs, fulfill social roles, contribute to formulating and executing social policies, and deter behaviors detrimental to the interests of the state and society. Ultimately, such participation contributes to social stability and the achievement of individual social integration [5]. According to some scholars, social participation revolves more around the interaction between public administration entities and the public [22]. Social participation serves as a pivotal element in achieving democracy and stands as a fundamental aspect of effective social governance and development. The theoretical framework delineates four modes of social participation: opinion expression, action organization, rights protection, and Internet-based engagement. In practice, social participation manifests in traditional and modern forms. Traditional methods include channels such as letters, visits, hearings, and opinion reflection. In contrast, modern participation involves various online platforms and networks facilitated through the internet [23, 24]. In specific studies on social participation, scholars have asserted a strong correlation between an individual’s level of social engagement and their health status. Additionally, comparative analyses have been conducted to examine disparities in social participation levels among diverse groups. Various factors influencing social participation encompass political, economic, societal, and cultural dimensions, among others [25, 26]. Social participation is significant as it reflects and influences individuals’ social status. It provides self-satisfaction, social integration, broadened perspectives, information access, enhanced personal skills, and fosters a sense of responsibility and national identity. Societally, it plays a crucial role in governance, boosting government trust, advancing social progress, and enhancing development vitality. It also helps reduce administrative costs and improve government efficiency [27]. As for how to promote the participation of civil society, it is suggested to start with public services, smooth channels for expression of opinions, and protection of citizens’ rights, so as to provide impetus and support for enhancing the participation level of civil society [28].

Youth social participation is a key way for young people to express their interests and impact society. Recognizing its significance, the government increasingly values youth engagement, which shapes national direction and future. Engaging youth in meaningful activities and decision-making processes addresses societal needs and extends their influence. In the late 1990s, youth social participation in the U.S. declined notably, with the elderly being more active during that period [29]. Similar to the scenario in the United States, Japan’s youth society is also grappling with a phenomenon termed as "low desire." There is an increasing number of Japanese young individuals who opt not to pursue marriage, parenthood, and exhibit a lack of interest in participating in social activities. This trend has significant implications and raises concerns about societal shifts and future demographic patterns within Japan [30]. Chinese youth groups, particularly the "post-90s" cohort, who constitute a significant proportion of the young population, have also displayed noticeable indications of "low desire" concerning social participation [31]. With the evolving times, the social participation of youth should advance towards greater independence, comprehensiveness, pragmatism, and diversity [32]. Some scholars categorize young people’s social participation into four aspects: transactional, rights protection, public welfare, and recreational participation [33]. The rise in young people’s social participation mirrors the elevation of their status in society. However, amid this surge, several issues have surfaced, including restricted participation, inadequate channels, irrational behaviors, a need for improved quality among young individuals, and a lag in the execution of social participation actions. Additionally, it’s crucial to address the willingness of rural young people to actively participate in society [34]. To foster youth social participation, government initiatives should focus on enhancing responsiveness, building trust in governance, meeting the genuine needs of young individuals, and ensuring institutional support. Utilizing publicity and education can be instrumental in augmenting the youth’s sense of social responsibility, political identity, and efficacy. This approach helps bolster their enthusiasm and engagement in social participation [35].

### Research hypothesis

On the whole, the academic community has extensively explored the significance of youth’s sense of gain and their pivotal role as active participants in society. This examination covers a broad spectrum of influencing factors, ranging from politics, economics, and social aspects to subjective perceptions and objective gains. However, this study distinguishes itself by focusing on measuring the level of youth’s social participation based on their sense of gain and further dissecting the impact mechanisms. Based on prevalent classification methods in current academia, this paper will define youth’s sense of gain through three perspectives: economic, political, and people’s livelihood [36]. The broad spectrum of social participation encompasses various dimensions, including political engagement, economic involvement, cultural immersion, social affairs participation, and numerous other facets [31]. This paper embraces a comprehensive understanding of youth social participation, analyzing it through three key facets: transactional engagement, rights protection, and public welfare initiatives [26, 37]. Various themes and value orientations prevalent in contemporary times significantly impact the motivation, content, and modes of youth’s social participation. These factors are closely intertwined with an individual’s perception of shared interests within society [38]. Community activity participation and service quality have a positive correlation with public satisfaction. Well-organized community activities and high-quality services contribute to increased public satisfaction [1]. Transactional social participation in affairs essentially involves citizens’ engagement in decision-making processes regarding public affairs. The level of activity in transactional social participation is often assessed by examining the correlation and alignment between individual interests and group interests. A higher sense of economic gain typically correlates with reduced reliance on group dependencies and lower activity levels in transactional social participation. The hypothesis proposed is as follows: The higher the sense of political acquisition among young individuals, leading to increased political consciousness and stronger group identity, correlates with heightened participation in transactional society. Additionally, a stronger sense of well-being among young people, coupled with substantial benefits from public services, acknowledgment of social justice, correlates with greater enthusiasm for transactional social participation. The proposed hypotheses are as follows:

H1: Youth’s sense of economic gain will have a significant negative impact on social participation in affairs;
H2: Youth’s sense of political gain will have a significant positive impact on social participation in affairs;
H3: Youth’s sense of livelihood gain will have a significant positive impact on social participation in affairs.

The sense of gain involves both horizontal (comparison with one’s past) and vertical (comparison with others) elements. When young individuals perceive their situation as relatively disadvantaged following these comparisons, they experience a psychological gap or a lower sense of fulfillment. This low sense of fulfillment prompts a heightened focus on resource allocation and personal rights. Consequently, they tend to maintain and advocate for their rights and interests through engagement in social participation activities aimed at rights protection [39, 40]. Therefore, the following hypothesis is proposed:

H4: Youth’s sense of economic gain will have a significant negative impact on social participation in rights protection;
H5: Youth’s sense of political gain will have a significant negative impact on social participation in rights protection;
H6: Young people’s sense of livelihood gain will have a significant negative impact on social participation in rights protection.

There is a positive correlation between individual values and subjective well-being. Public welfare social participation can bring youth a sense of achievement and subjective well-being, thus increasing their enthusiasm for participation. Public welfare activities also have a positive correlation with happiness [1]. Therefore, the following hypothesis is proposed:

H7: Youth’s sense of economic gain will have a significant positive impact on social participation in public welfare;
H8: Youth’s sense of political gain will have a significant positive impact on social participation in public welfare;
H9: Youth’s sense of well-being will have a significant positive impact on social participation in public welfare.

This paper utilizes the nine hypotheses regarding youth’s sense of gain as a focal point. It categorizes social participation into three dimensions: transactional, rights protection, and public welfare participation. Through a detailed analysis, it specifically explores how youth’s sense of gain influences various forms of social participation. Moreover, based on empirical research findings, the paper presents policy recommendations. These recommendations serve as essential references for the government to enhance youth’s sense of gain and promote their active participation in society.

## Materials and methods

### Methods

The empirical analysis in this paper is based on data from the Chinese Social Survey (CSS), a large-scale continuous sampling survey initiated by the Institute of Sociology of the Chinese Academy of Social Sciences in 2005. Conducted biennially nationwide, CSS aims to capture data on social changes in China during the transition period. For the purposes of this paper, the CSS2021 database is chosen, encompassing 592 village neighborhood committees in 30 provinces/municipalities/autonomous regions throughout the country. The specific model operation involves selecting working youth as the empirical sample. The working youth demographic, characterized by relatively stable economic income and a well-established social security system, possesses formal avenues for engagement in social affairs—attributes aligning with the pertinent variables and questions in this study. Adhering to the definition of young individuals aged 14 to 35 as stipulated in the Chinese Medium and Long Term Youth Development Plan (2016–2025) and considering the minimum employment age of 16 years mandated by China’s labor law, this paper selects a sample of working young people aged between 16 and 35. After eliminating missing values, the sample size consists of 1,014 individuals.

### Ethics statement

This study utilizes publicly available data from the CSS2021 database, which is freely accessible and provided by the Chinese Social Survey platform. As this study involves secondary data analysis of anonymized, publicly accessible information, it does not require Institutional Review Board (IRB) or ethics committee approval. Informed written or verbal consent was not obtained, as the data used are de-identified and freely available for public use.

### Variable design

#### Dependent variables

According to the current research and the previous definition of social participation, this paper defines social participation from three dimensions: transactional social participation, rights protection social participation and public welfare social participation.

This study assesses youth social participation in affairs using the H2a question from the CSS2021 questionnaire. It includes activities like reporting social issues, participating in policy meetings, attending hearings, and engaging in decision-making.

This study assesses youth social participation in safeguarding rights using the H2a question from the CSS2021 questionnaire. It covers activities like expressing opinions to government departments, voicing views on policies, petitioning, and participating in collective rights actions.

This study examines youth social participation in public welfare using multiple questions from the CSS2021 questionnaire. It includes participation in spontaneous public welfare organizations (H1a), community or self-organized activities in the past two years (H2a), and volunteer services in the past year (I1a).

Participants are assigned a value of 1, while non-participants receive 0. "None of the above" responses are treated as missing and converted to 0 for analysis. See Table 1 for details.

**Table 1:** Dependent variable definition and operational summary.

Property	Name	Operationalization	Assign
**Dependent Variables**	**Affairs**	**H2a. In the last two years, have you been involved in any of the following?** 2. Reflecting social problems to newspapers, radio stations, Internet forums, and other medias; 3. Contribute professional knowledge in public policy and affairs meetings; 4. Attending public policy hearings organized by government departments; 5. Participating in major decision-making discussions in the village/unit.	Yes = 1/ No = 0
	**Rights** **Protection**	**H2a. In the last two years, have you been involved in any of the following?** 3. To reflect opinions to government departments (including by telephone, email, etc.); 5. Express personal opinions on government policies through various channels; 7. Go to government departments to petition; 11. Participate in online/offline collective rights protection actions.	Yes = 1/ No = 0
	**Public Welfare**	**H1a. Which of the following groups are you currently a member of?** 5. Voluntary public welfare organizations (such as volunteer groups, owners’ committees, etc.). **H2a. In the last two years, have you been involved in any of the following?** 9. Engage in community or self-organized social welfare activities, like volunteering for blood donation, environmental cleanup, or assisting the elderly, disabled, and sick. **I1a. Which of the following volunteer services have you personally participated in in the past year?** 1. Child care; 2. Youth counseling; 3. Elderly care; 4. Women’s rights/protection; 5. Help the disabled; 6. Volunteer teaching assistant; 7. Help the poor; 8. Medical care; 9. Legal aid; 10. Environmental protection; 11. Rescue and disaster relief; 12. International assistance; 13. Other	Yes = 1/ No = 0

### Independent variables

This study assesses youth’s sense of economic gain through their employment situation and income distribution. It utilizes the F3a sub-topic from the CSS2021 questionnaire, which addresses perceptions of fairness in work and income distribution. Additionally, it incorporates the G3a sub-section on government performance in economic development. The cumulative scores from these questions represent the youth’s sense of economic gain.

This study evaluates youth’s sense of political gain through perceptions of political rights, government responsiveness, and societal stability. It draws on the F3a sub-topic of the CSS2021 questionnaire, which focuses on the fairness of political rights. Additionally, it includes the G3a sub-section on government performance in political rights, crime control, social order, transparency, and responsiveness. The combined scores from these questions represent the youth’s sense of political gain.

This study evaluates young people’s sense of livelihood gain through medical care, social security, and public services. It utilizes the F3a sub-topic from the CSS2021 questionnaire, which focuses on fairness in public healthcare. Additionally, it incorporates G3a questions related to government performance in health services, social security, and environmental protection. The combined scores from these questions represent the youth’s sense of livelihood gain. See Table 2 for details.

**Table 2:** Independent variable definition and operational summary.

Property	Name	Operationalization	Assign
**Independent Variables**	**Economic Gain**	**F3a. How fair do you think the following aspects of social life are today?** 5. Jobs and employment opportunities; 6. Distribution of wealth and income **G3a. Do you think the government is doing a good job in the following areas?** 8. To develop the economy and increase people’s income; 9. Expand employment and increase employment opportunities	It’s hard to say = -1; Very unfair/very bad = 1; Not very fair/not very good = 2; Fair/good = 3; Very fair/very good = 4; Aggregate score
	**Political Gain**	**F3a. How fair do you think the following aspects of social life are today?** 2. The political rights that citizens actually enjoy. **G3a.Do you think the government is doing a good job in the following areas?** 4. Guarantee the political rights of citizens; 5. Fight crime and maintain social order; 10. Enhance government transparency by opening government information. 11. Responsive to public demands with a service-oriented attitude	
	**Livelihood Gain**	**F3a. How fair do you think the following aspects of social life are today?** 4. Public health care **Do you think the government is doing a good job in the following areas?** 1. Provision of health services; 2. To provide social security for the masses; 3. Protect the environment and control pollution; 13. Ensure food and drug safety; 14. Promote and expand cultural and sports activities.	

### Control variables

This paper aims to mitigate model result discrepancies stemming from missing variables and to ensure minimal interference with research conclusions. Building on existing research on factors influencing perception of gain, this paper redefines variables and incorporates additional control variables in the empirical analysis. The control variables considered in this paper are: Age; Gender; Political status; Education; Income, according to the American statistician William Corrente’s "rough score method" to re-process the income level data, income is divided into 5 equal points; Government trust, this paper draws upon scholar work on government trust and employs a segment of question F1a from the CSS2021 questionnaire [41]. See Tables 3–5 for details.

**Table 3:** Control variable definition and operational summary.

Property	Name	Operationalization	Assign
**Control Variables**	**Age**	**a1c1. Your year of birth?**	2021- year of birth
	**Gender**	**a1b1. What is your gender?**	Male = 1/Female = 0
	**Education**	**a1d1. What is your education level?**	Never went to school = 1; Primary school = 2; Junior high school = 3; Senior high school = 4; Junior college = 5; Bachelor degree or above = 6
	**Political**	**A3. What is your political affiliation?**	Party membership = 1/Non = 0;
	**Income**	**B11a. What are your various incomes?** 1. Gross income	Below 10,000 = 1; 10,000–30,000 = 2; 30,000–50,000 = 3; 50,000–100,000 = 4; More than 100,000 = 5
	**Government Trust**	**F1a. Do you trust the following organizations?** 1. The Central Government; 2. District and county governments; 3. Township governments; 9. Courts of law; 10. Public security Department	It’s hard to say = -1; Very distrust = 1; Not very trust = 2; Trust = 3; Very trust = 4; Aggregate score

**Table 4:** Description of control variables 1.

Variable	N	Mean	SD	Min	Max
Age	1,014	29.64	3.926	20	35
Gender	1,014	0.514	0.499	0	1
Political	1,014	0.125	0.341	0	1
Education	1,014	4.465	1.253	1	6
Income	1,014	3.548	1.163	1	5
Trust	1,014	15.89	3.427	-5	20

**Table 5:** Description of control variables 2.

Variable	Group	Scale	Variable	Group	Scale
Age	20–25 26–30 31–35	14.1% 36.7% 49.2%	Education	Never went to school Primary school Junior high school Senior high school Junior college Bachelor degree or above	0.8% 3.4% 21.1% 10.9% 35.7% 28.2%
Gender	Male Female	51.4% 48.6%			
Work	Yes No	100% 0%	Political	Yes No	12.5%
					87.5%

### Model design

The logistic model, utilized due to the categorical nature of the dependent variable, social participation, is based on natural variables. It’s a binary logistic regression model formulated as follows:

$$Y_{1-3}=\ln(\frac{p}{1-p})=\beta_{0}+\beta_{1}x_{1}+\beta_{2}x_{2}+\beta_{3}x_{3}+\sum Controls+\varepsilon$$ (1)

The formula denotes *y*_*1*_*-y*_*3*_ as representing youth’s transactional, rights protection, and public welfare social participation, while *x*_*1*_*-x*_*3*_ symbolize youth’s sense of economic gain, political gain, and livelihood gain, respectively. *ΣControls* signify control variables. *β*_*0*_ and *ε* signify constant and random error terms.

Before conducting the regression analysis, a multicollinearity test was performed on both the dependent and independent variables. The test results revealed values between 0 and 2 for the Variance Inflation Factor (VIF) for each variable, indicating an absence of multicollinearity issues within the constructed model.

The reliability of the scale for youth’s sense of gain was assessed using Cronbach’s α, and the obtained result of α = 0.946 indicated high reliability within this scale. To further validate the scale, the KMO sampling appropriateness measure and Bartlett sphericity were employed to test the suitability of the measurement scale for factor analysis.

For the validity test of the scale measuring youth’s sense of economic gain, the KMO measure was 0.635, surpassing the 0.6 threshold, and Bartlett’s test was significant at 0.000, indicating strong correlations suitable for factor analysis. The exploratory factor analysis, using principal component analysis, showed a cumulative variance of 59.608%, with all factor loadings above 0.5, demonstrating that the selected items effectively represented the youth’s sense of economic gain. The obtained factor component matrix is shown in Table 6.

**Table 6:** Principal component factor analysis of economic sense of gain.

Item	Factor load
To develop the economy and increase people’s income	0.816
Expand employment and increase employment opportunities	0.806
Wealth and income distribution	0.746
Jobs and employment opportunities	0.715
Explained variance	59.608%
observations	1,014

For the validity test of the scale measuring youth’s political gain, the KMO measure was 0.832, surpassing the 0.6 threshold, and Bartlett’s test was significant at 0.000, indicating strong correlations suitable for factor analysis. The exploratory factor analysis revealed a cumulative variance of 62.795%, with all factor loadings above 0.5, demonstrating that the selected items effectively represented the youth’s sense of political gain. The obtained factor component matrix is shown in Table 7.

**Table 7:** Principal component factor analysis of political sense of gain.

Item	Factor load
Enhance government transparency by opening government information	0.849
Responsive to public demands with a service-oriented attitude	0.840
Guarantee the political rights of citizens	0.834
Fight crime and maintain social order	0.788
The political rights that citizens actually enjoy	0.630
Explained variance	62.795%
Observations	1,014

The validity test for the scale measuring youth’s sense of livelihood gain showed a KMO measure of 0.874, exceeding 0.6, and Bartlett’s test was significant at 0.000, indicating strong correlations suitable for factor analysis. The exploratory factor analysis revealed a cumulative variance of 62.103%, with all factor loadings above 0.5, confirming that the selected items effectively represented the youth’s sense of livelihood gain. The obtained factor component matrix is shown in Table 8.

**Table 8:** Principal component factor analysis of people’s livelihood sense of gain.

Item	Factor load
To provide social security for the masses	0.864
Provision of health services	0.845
Promote and expand cultural and sports activities	0.835
Ensure food and drug safety;	0.833
Protect the environment and control pollution	0.761
Public health care	0.544
Explained variance	62.103%
Observations	1,014

## Statistical analysis

### Descriptive analysis

#### Descriptive analysis of youth’s sense of gain

Overall, the youth group exhibits the highest level of satisfaction in terms of people’s livelihood, with a mean value of 17.69 and a standard deviation of 3.851. The second-highest level is observed in the sense of political gain, with a mean of 14.61 and a standard deviation of 3.560. In contrast, the sense of economic gain registers the lowest level among the three dimensions, with a mean of 11.04 and a standard deviation of 2.695. See Table 9 for details.

**Table 9:** Descriptive analysis of youth’s sense of gain.

Sense of gain	N	Mean	SD	Min	Max
Economic gain	1,014	11.04	2.695	-4	16
Political gain	1,014	14.61	3.560	-5	20
Livelihood gain	1,014	17.69	3.851	-6	24

Descriptive analysis of each component of youth sense of economic gain. Understanding the sense of economic gain focuses on evaluating two primary aspects: youth employment and income distribution. 73.3% perceive the government’s job opportunities handling as fairly fair, with 25.3% expressing dissatisfaction. Regarding employment enhancement efforts, 74.9% acknowledge the government’s work as good or very good, indicating a high level of approval. In terms of wealth and income distribution, 63.4% find it fair or very fair, yet 34.8% disagree. However, 75.5% praise the government’s efforts in economic development and income increase as good or very good, showing widespread acknowledgment and positive economic gain perception regarding income growth among young people. See Table 10 for details.

**Table 10:** Descriptive analysis of economic gain.

Variable	Item	Option	N	Percent
**Economic Gain**	Jobs and employment opportunities	It’s hard to say = -1 Very unfair = 1 Not very fair = 2 Fair = 3 Very fair = 4	15 36 243 675 135	1.4% 3.3% 22.0% 61.1% 12.2%
	Expand employment and increase	It’s hard to say = -1 Very bad = 1 Not very good = 2 Good = 3 Very good = 4	28 32 217 651 176	2.5% 2.9% 19.7% 59.0% 15.9%
	Wealth and income distribution	It’s hard to say = -1 Very unfair = 1 Not very fair = 2 Fair = 3 Very fair = 4	19 70 315 581 119	1.7% 6.3% 28.5% 52.6% 10.8%
	To develop the economy and increase people’s income	It’s hard to say = -1 Very bad = 1 Not very good = 2 Good = 3 Very good = 4	23 33 214 653 181	2.1% 3.0% 19.4% 59.1% 16.4%

Descriptive analysis of each component of youth sense of political gain. Youth’s political sense of gain involves perceptions of political rights, social stability, and government responsiveness. To delve into their political gain, it’s notable that 80% of young individuals acknowledge their perceived political rights as fairly fair, while 85.4% recognize the government’s efforts in ensuring citizens’ political rights. Furthermore, 77.4% acknowledge the government’s role in maintaining social order, signaling a high sense of achievement in social stability. Additionally, over 75% appreciate the government’s transparency efforts in disclosing information and its responsive nature to public demands, contributing to a sense of gain among the youth regarding government responsiveness. See Table 11 for details.

**Table 11:** Descriptive analysis of political gain.

Variable	Item	Option	N	Percent
**Political Gain**	The political rights that citizens actually enjoy.	It’s hard to say = -1 Very unfair = 1 Not very fair = 2 Fair = 3 Very fair = 4	23 43 155 629 254	2.1% 3.9% 14.0% 57.0% 23.0%
	Guarantee the political rights of citizens;	It’s hard to say = -1 Very bad = 1 Not very good = 2 Good = 3 Very good = 4	32 21 108 755 188	2.9% 1.9% 9.8% 68.4% 17.0%
	Fight crime and maintain social order;	It’s hard to say = -1 Very bad = 1 Not very good = 2 Good = 3 Very good = 4	40 32 178 649 205	3.6% 2.9% 16.1% 58.8% 18.6%
	Enhance government transparency by opening government information.	It’s hard to say = -1 Very bad = 1 Not very good = 2 Good = 3 Very good = 4	40 32 178 649 205	3.6% 2.9% 16.1% 58.8% 18.6%
	Responsive to public demands with a service-oriented attitude	It’s hard to say = -1 Very bad = 1 Not very good = 2 Good = 3 Very good = 4	29 27 216 654 178	2.6% 2.4% 19.6% 59.2% 16.1%

Descriptive analysis of each component of sense of youth’s livelihood gain. The assessment of people’s livelihood encompasses health care, social security, and public services. Regarding health care and social security, over 85% of young individuals perceive fairness in public medical care and believe the government’s efforts in these domains are commendable or highly commendable, indicating a strong sense of access to medical care and social security. Concerning public services, the assessment revolves around the government’s endeavors in "environmental protection and pollution control," "ensuring food and drug safety," and "enhancing cultural and sports activities, fostering cultural and sports programs." More than 80% of young people acknowledge the government’s efficacy across these areas, reflecting a robust sense of fulfillment in public services. See Table 12 for details.

**Table 12:** Descriptive analysis of livelihood gain.

Variable	Item	Option	N	Percent
**Livelihood Gain**	Public health care	It’s hard to say = -1 Very unfair = 1 Not very fair = 2 Fair = 3 Very fair = 4	13 19 138 728 206	1.2% 1.7% 12.5% 65.9% 18.7%
	Provision of health services	It’s hard to say = -1 Very bad = 1 Not very good = 2 Good = 3 Very good = 4	21 12 108 784 179	1.9% 1.1% 9.8% 71.0% 16.2%
	To provide social security for the masses	It’s hard to say = -1 Very bad = 1 Not very good = 2 Good = 3 Very good = 4	26 14 137 760 167	2.4% 1.3% 12.4% 68.8% 15.1%
	Protect the environment and control pollution	It’s hard to say = -1 Very bad = 1 Not very good = 2 Good = 3 Very good = 4	17 35 182 688 182	1.5% 3.2% 16.5% 62.3% 16.5%
	Ensure food and drug safety	It’s hard to say = -1 Very bad = 1 Not very good = 2 Good = 3 Very good = 4	30 11 96 744 223	2.7% 1.0% 8.7% 67.4% 20.2%
	Promote and expand cultural and sports activities	It’s hard to say = -1 Very bad = 1 Not very good = 2 Good = 3 Very good = 4	25 11 117 740 211	2.3% 1.0% 10.6% 67.0% 19.1%

### Descriptive analysis of youth social participation

The analysis and statistical comparison of youth social participation reveal a hierarchical order: public welfare (26%) > right protection (13.8%) > affair (12%). This comparison highlights the lower overall involvement of young individuals in various societal engagements, accentuating the consistent necessity to strengthen and promote heightened levels of social participation among the youth. It emphasizes the ongoing requirement to encourage and foster increased engagement and active contributions within society among this demographic. See Table 13 for details.

**Table 13:** Descriptive analysis of social participation.

Variable	Item	Option	N	Percent
**Affairs**	**H2a. In the last two years, have you been involved in any of the following?** 2. Reflecting social problems to newspapers, radio stations, Internet forums, and other medias; 3. Contribute professional knowledge in public policy and affairs meetings; 4. Attending public policy hearings organized by government departments; 5. Participating in major decision-making discussions in the village/unit.	Yes No	152 952	13.8% 86.2%
**Rights** **Protection**	**H2a. In the last two years, have you been involved in any of the following?** 3. To reflect opinions to government departments (including by telephone, email, etc.); 5. Express personal opinions on government policies through various channels; 7. Go to government departments to petition; 11. Participate in online/offline collective rights protection actions.	Yes No	133 971	12% 88%
**Public Welfare**	**H1a. Which of the following groups are you currently a member of?** 5. Voluntary public welfare organizations (such as volunteer groups, owners’ committees, etc.). **H2a. In the last two years, have you been involved in any of the following?** 9. Engage in community or self-organized social welfare activities, like volunteering for blood donation, environmental cleanup, or assisting the elderly, disabled, and sick. **I1a. Which of the following volunteer services have you personally participated in in the past year?** 1. Child care; 2. Youth counseling; 3. Elderly care; 4. Women’s rights/protection; 5. Help the disabled; 6. Volunteer teaching assistant; 7. Help the poor; 8. Medical care; 9. Legal aid; 10. Environmental protection; 11. Rescue and disaster relief; 12. International assistance; 13. Other	Yes No	287 817	26% 74%

### Correlation analysis

The correlation analysis conducted on independent, dependent, and control variables is presented in Table 14 offering initial insights for hypothesis testing. The results indicate that within the independent variables, concerning the relationship between the sense of gain and social participation in affairs, there’s a negative correlation between youth’s sense of economic gain and transactional social participation, but it lacks statistical significance. Thus, H1 has not been preliminarily supported. Additionally, while there’s a positive correlation between youth’s sense of political gain and transactional social participation, it is not statistically significant, failing to verify H2. Furthermore, the positive correlation observed between youth’s sense of livelihood and transactional social participation also lacks statistical significance, thereby not supporting H3 at this preliminary stage.

**Table 14:** Correlation analysis.

Variable	Affair	Welfare	Rights	Economy	Political	Livelihood
Affair	1					
Welfare	0.189**	1				
Rights	0.304**	0.079**	1			
Economic	0.048	0.025	0.107**	1		
Political	0.013	0.076*	0.096**	0.749**	1	
Livelihood	0.010	0.052	0.064*	0.733**	0.846**	1
Gender	0.020	0.001	0.009	0.035	0.057	0.068*
Age	0.014	0.022	0.010	0.056	0.088**	0.074*
Education	0.081**	0.149**	0.072*	0.022	0.115**	0.084**
Political	0.119**	0.088**	0.045	0.056	0.112**	0.091**
Income	0.042	0.032	0.077*	0.004	0.042	0.020
Trust	0.009	0.064*	0.092**	0.406**	0.503**	0.462**

Regarding the correlation between the sense of gain and social participation in rights protection, notable findings emerged. There exists a significant negative correlation between the sense of economic gain and youth’s engagement in rights protection activities, indicating preliminary support for H4. Similarly, a significant negative correlation was observed between young people’s sense of political gain and their involvement in rights protection activities, preliminarily verifying H5. Additionally, a negative correlation between youth’s sense of livelihood and rights protection was found, offering preliminary support for H6.

In examining the correlation between the sense of gain and social participation in public welfare, some interesting trends emerged. Although the sense of economic gain among youth showed a positive correlation with public welfare social participation, it did not achieve statistical significance, leading to the non-verification of H7. Conversely, a positive correlation was observed between young people’s sense of political gain and their engagement in public welfare activities, preliminarily confirming H8. However, the positive correlation between youth’s sense of well-being and public welfare social participation did not attain statistical significance, resulting in the non-verification of H9.

Regarding the control variables in Table 15, the analysis highlighted substantial positive correlations between education level and transactional, public welfare, and rights protection social participation. Additionally, political status exhibited positive correlations with transactional and public welfare social participation. Notably, income showed a significant positive correlation with rights protection social participation. Moreover, government trust displayed a significant positive correlation with public welfare social participation, while revealing a noteworthy negative correlation with rights protection social participation.

**Table 15:** Correlation analysis of control variables.

Variable	Sex	age	Education	Political	Income	Trust
Gender	1					
Age	0.019	1				
Education	0.110**	0.099**	1			
Political	0.027	0.058	0.279**	1		
Income	0.237**	0.139**	0.217**	0.092**	1	
Trust	0.064*	0.104**	0.184**	0.132**	0.034	1

Note

* means p<0.05 (double tail)

**means p<0.01 (double tail)

### Regression analysis

This paper constructs a binary logistic regression model to analyze the impact of youth’s sense of acquisition on social participation. The regression results are analyzed from three aspects: transactional, rights protection social participation and public welfare social participation.

### Youth social participation in affairs

From Table 16, Model 1 exclusively includes control variables. According to the regression outcomes, the education level of youth displays a significantly positive correlation with transactional social participation, suggesting that higher educational attainment corresponds to increased engagement in transactional social participation activities. Furthermore, a significant positive correlation emerges between the political status of youth and transactional social participation, with the likelihood of engagement being 1.059 times higher for young Communist Party members compared to non-party members. This underscores the influential role of political affiliations, aligning with prior findings that the political influence of party groups significantly impacts the social participation of young individuals.

**Table 16:** Regression results of youth’s sense of gain and transactional social participation.

Variable	Model 1				Model 2			
	Coef.	Exp(B)	Sig.	SE.	Coef.	Exp(B)	Sig.	SE.
Economic					0.129	0.914	0.008**	0.879
Political					0.049	0.921	0.366	1.050
Livelihood					0.031	1.080	0.508	1.032
Gender	0.167	1.182	0.367	0.186	0.149	1.070	0.424	1.160
Age	0.008	1.008	0.742	0.024	0.009	0.991	0.716	1.009
Education	0.147	1.158	0.08*	0.084	0.129	1.213	0.127	1.138
Political	0.722	2.059	0.002**	0.236	0.708	1.374	0.003**	2.029
Income	0.026	1.027	0.697	0.067	0.026	1.162	0.698	1.027
Trust	0.015	0.985	0.574	0.027	0.013	0.935	0.690	0.987
Constant	0.167	0.060	0.003	0.960	2.653	0.248	0.009	0.070
Observations	1,014				1,014			

Note

*means p<0.1

**means p<0.05

In Model 2, incorporating political, economic, and livelihood factors for regression analysis, the results reveal a significant negative correlation between the sense of economic gain among youth and transactional social participation, thereby confirming H1. This aligns with earlier research conclusions that compared political participation behaviors across different income groups, indicating a tendency for groups with higher economic status to participate less in community affairs [42]. The empirical findings in this study similarly suggest that young individuals with a heightened sense of economic gain exhibit reduced attention towards public affairs. The empirical results of this paper also show that young people with higher sense of economic gain pay less attention to public affairs.

### Youth social participation in rights protection

From Table 17, Model 3 solely includes control variables. As per the regression outcomes, the education level among young individuals exhibits a significantly positive correlation with social participation in rights protection, suggesting that higher education is linked to increased awareness among youth regarding safeguarding their rights. Moreover, there’s a noteworthy positive correlation between the income of youth and their engagement in rights protection activities, indicating that higher-income groups are more inclined to participate in such endeavors. Conversely, a significant negative correlation surfaces between government trust and rights protection, signifying that lower levels of trust in the government correspond to increased participation in rights protection activities among young people.

**Table 17:** Regression results of youth’s sense of gain and social participation in rights protection.

Variable	Model 3				Model 4			
	Coef.	Exp(B)	Sig.	SE.	Coef.	Exp(B)	Sig.	SE.
Economic					0.090	0.914	0.079*	0.051
Political					0.082	0.921	0.105	0.050
Livelihood					0.077	1.080	0.089*	0.045
Gender	0.077	0.196	0.697	1.080	0.068	1.070	0.730	0.197
Age	0.006	0.026	0.819	0.994	0.010	0.991	0.719	0.026
Educational	0.195	0.090	0.030**	1.215	0.193	1.213	0.033**	0.091
Political	0.292	0.271	0.281	1.339	0.317	1.374	0.246	0.273
Income	0.141	0.073	0.054*	1.152	0.150	1.162	0.041**	0.074
Trust	0.097	0.026	0.000**	0.907	0.067	0.935	0.027**	0.030
Constant	1.784	0.997	0.074	0.168	1.394	0.248	0.183	1.048
Observations	1,014				1,014			

Note

* means p<0.1

** means p<0.05

Moving to Model 4, which integrates political, economic, and livelihood factors for regression analysis, the results unveil a significant negative correlation between the sense of economic gain and rights protection, verifying H4. This implies that youth with a lower sense of economic gain are more inclined to protect their rights and interests by actively engaging in rights protection activities. Moreover, there’s a significant positive correlation between the sense of livelihood gain and rights protection, confirming H6. This suggests that heightened awareness of livelihood gain prompts greater attention towards related benefits and a heightened willingness to engage in rights protection activities.

### Youth social participation in public welfare

Based on Table 18, Model 5 comprised solely control variables. The regression outcomes indicate a noteworthy positive correlation between the education level of youth and their involvement in public welfare social participation. This suggests that higher education levels are associated with a greater inclination toward participating in public welfare activities. Transitioning to Model 6, which incorporates political, economic, and livelihood factors for regression analysis, the findings highlight a significant positive correlation between the sense of political gain and public welfare social participation, affirming H8. This outcome implies that young individuals with a heightened sense of political gain tend to possess greater political awareness and consciousness, displaying a heightened willingness to engage in public welfare activities. Their contributions to society align with a pursuit of self-fulfillment and social integration, aligning with previous research conclusions by scholars.

**Table 18:** Regression results of youth’s sense of gain and public welfare social participation.

Variable	Model 5				Model 6			
	Coef.	Exp(B)	Sig.	SE.	Coef.	Exp(B)	Sig.	SE
Economic					0.043	0.958	0.295	0.958
Political					0.081	1.084	0.068*	1.084
Livelihood					0.017	0.983	0.660	0.983
Gender	0.094	1.099	0.520	0.146	0.086	1.090	0.558	1.090
Age	0.024	1.025	0.210	0.019	0.027	1.027	0.172	1.027
Education	0.279	1.321	0.000**	0.066	0.273	1.314	0.000	1.314
Political	0.252	1.286	0.216	0.204	0.225	1.253	0.270	1.253
Income	0.022	0.978	0.671	0.053	0.026	0.975	0.625	0.975
Trust	0.026	1.027	0.235	0.022	0.008	1.008	0.761	1.008
Constant	3.523	0.030	0.000	0.774	3.670	0.025	0.000	0.025
Observations	1,014				1,014			

Note:

* means p<0.1

** means p<0.05

## Results and discussion

Based on CSS2021 data, the scales measuring the sense of economic gain, sense of political gain, and sense of people’s livelihood gain demonstrated both reliability and validity. Correlation analyses revealed several key findings: a lower sense of economic gain is significantly negatively correlated with social participation in rights protection and general social participation. This aligns with ’’social exchange theory’’, which posits that individuals are more likely to engage in social activities when the perceived benefits outweigh the costs. Additionally, the sense of political gain is significantly positively correlated with public welfare social participation but negatively correlated with general social participation. This supports ’’social capital theory’’, which suggests that engagement in specific social activities can enhance individual networks and overall well-being. Conversely, the sense of people’s livelihood gain is significantly negatively correlated with social participation, which is consistent with ’’theories of civic engagement’’ that argue a strong sense of well-being fosters increased commitment to social activities.

Regression analysis using a binary logistic model confirmed these relationships and validated the following hypotheses: Hypothesis 1 is valid, youth’s sense of economic gain will have a significant negative impact on social participation in affairs. Hypothesis 4 is valid, as the sense of economic gain is significantly negatively correlated with rights protection activities, indicating that lower economic gain leads to higher participation in rights protection. Hypothesis 6 is valid, with a significant positive correlation between the sense of people’s livelihood gain and participation in rights protection activities, suggesting that higher livelihood gain encourages greater involvement in such activities. Hypothesis 8 is also valid, showing a significant positive correlation between the sense of political gain and public welfare social participation, meaning that a higher sense of political gain increases participation in public welfare activities. Additionally, control variables such as education level, political outlook, and government trust significantly affect youth’s social participation. These findings offer a nuanced understanding of how various dimensions of gain influence different forms of social participation, supported by relevant theoretical frameworks. A summary is given in Table 19.

**Table 19:** Summary of hypothesis test results.

Research hypothesis	Result
**H1:** Youth’s sense of economic gain will have a significant negative impact on social participation in affairs.	**Set up**
**H2:** Youth’s sense of political gain will have a significant positive impact on social participation in affairs.	False
**H3:** Youth’s sense of livelihood gain has a significant positive impact on transactional social participation.	False
**H4:** Youth’s sense of economic gain will have a significant negative impact on social participation in in rights protection.	**Set up**
**H5:** Youth’s sense of political gain will have a significant negative impact on social participation in in rights protection.	False
**H6:** Young’s sense of livelihood gain will have a significant negative impact on social participation in rights protection.	**Set up**
**H7:** Youth’s sense of economic gain will have a significant positive impact on public welfare social participation.	False
**H8:** Youth’s sense of political gain will have a significant positive impact on public welfare social participation.	**Set up**
**H9:** Youth’s sense of livelihood gain will have a significant positive impact on public welfare social participation.	False

## Conclusions and policy implications

This study, based on the CSS2021 dataset and a sample of 1,014 employed young individuals, explores the relationship between youth’s sense of gain in economic, political, and livelihood dimensions and their social participation. The analysis reveals that a higher sense of economic gain correlates negatively with transactional social participation, indicating that youth with strong economic aspirations and desires for upward mobility may see limited tangible benefits in engaging in social affairs. These individuals, often beneficiaries of economic development, tend to prioritize socio-political stability, adhere to existing norms, and show limited interest in public affairs or political engagement [31]. To address these findings, policies should focus on creating targeted opportunities for social participation that align with young people’s economic aspirations. For instance, establishing programs that link civic engagement with career development—such as internships or volunteer positions that offer professional networking and skill-building—can encourage greater involvement. Additionally, implementing initiatives that clearly demonstrate the tangible benefits of social participation, like showcasing success stories of individuals whose engagement has led to career advancement or community impact, can help bridge the gap between youth aspirations and their engagement in public affairs. These steps can make social participation more relevant and appealing to young people, thereby increasing their involvement.

Secondly, examining social participation in rights protection reveals that a higher sense of economic gain is negatively correlated with engagement in rights protection, while a strong sense of livelihood gain shows a positive correlation. Youth with lower economic satisfaction may feel relatively deprived, leading to negative sentiments and motivating their involvement in rights protection, sometimes through non-institutionalized means. This supports previous research suggesting that inadequate social governance and slow governmental responses can drive young people toward more irrational and impassioned forms of social participation [35]. Youth with higher economic gain tend to approach rights protection more rationally, weighing costs and benefits, while those with a stronger sense of livelihood gain are more motivated to protect their rights, especially regarding public services. To address these dynamics, policies should focus on ensuring timely and equitable access to essential public services such as healthcare and social security, which will help reduce feelings of deprivation among youth. Additionally, targeted educational campaigns should promote awareness of rights protection, providing practical information on how to engage effectively. Developing user-friendly platforms for accessing information and participating in rights protection activities can further support these efforts. These measures can help foster a more informed and proactive approach to rights protection among all youth groups.

Thirdly, considering public welfare social participation, there exists a noteworthy positive correlation between the sense of political gain and involvement in such activities. This reaffirms prior research that highlights the influence of political engagement, values, satisfaction with social life, and trust in society on the participation levels of youth [20]. In accordance with Maslow’s hierarchy of needs theory, young individuals, as social beings, possess not just fundamental survival needs but also the desire for self-realization. Engaging in public welfare activities offers a means for young people to fulfill these needs. Nevertheless, there’s a prevalent lack of independent consciousness among young individuals when it comes to voluntary service participation, leading to a higher dependence on external organizations for such initiatives [43]. Given the limited involvement of Chinese youth in public welfare activities, advancing the culture of volunteerism and enhancing volunteer service infrastructure is crucial. To address this, integrating volunteerism into educational curricula can instill a sense of social responsibility from an early age. Developing local and national volunteer networks and online platforms can help connect youth with public welfare initiatives. Additionally, investing in training programs and resource centers will support volunteer activities, while recognizing and rewarding contributions can further motivate young people to engage. These measures can help foster a more vibrant culture of volunteerism and encourage greater youth participation in public welfare.

On the whole, it is crucial for the government to recognize the importance of youth’s sense of gain in fostering social participation. Enhancing this sense of gain can strengthen the relationship between youth and the government, creating a solid foundation for mutual trust and engagement. To achieve this, the government should develop innovative mechanisms that address the real interests and needs of young people. Legislative measures can be implemented to protect their participation rights, foster a sense of social responsibility, and encourage their active involvement in public welfare. Additionally, strengthening volunteer service infrastructure and promoting a culture of volunteerism are key steps in boosting youth engagement in social participation [20]. Establishing standardized and institutionalized platforms for youth to engage in public affairs and voice their opinions is crucial. These platforms can guide young people towards participating within structured systems, reducing the risk of negative societal repercussions from demands expressed outside these channels. Strengthening educational campaigns and public outreach is also essential to enhance youth awareness and involvement in social participation. This includes emphasizing the importance of such engagement and developing their skills and capacity for active participation. Additionally, adopting an elitist approach to highlight positive role models can steer youth towards constructive social participation.

## Limitations

Acknowledging a study’s limitations is crucial for its credibility and future research. This study offers valuable insights into the link between youth’s sense of gain and social participation but has several limitations. First, the findings are based on a specific sample of working youth in China, which may limit their generalizability to other populations. Cultural and contextual factors unique to China might affect the applicability of these results elsewhere. Future research should address these limitations by including diverse demographic groups and international contexts. Comparative studies across cultures could enhance external validity. Employing mixed-methods approaches, such as qualitative interviews or focus groups, could provide deeper insights. Longitudinal studies might capture changes over time, offering a richer understanding of how youth perspectives and societal contexts evolve. We aim to pursue a more comprehensive and interdisciplinary approach to better understand the complex relationship between youth’s sense of gain and their social participation.
